# In vivo characteristics of targeted drug-carrying filamentous bacteriophage nanomedicines

**DOI:** 10.1186/1477-3155-9-58

**Published:** 2011-12-20

**Authors:** Lilach Vaks, Itai Benhar

**Affiliations:** 1 Department of Molecular Microbiology and Biotechnology, The George S. Wise Faculty of Life Sciences, Tel-Aviv University, Ramat Aviv, Israel

## Abstract

**Background:**

Targeted drug-carrying phage nanomedicines are a new class of nanomedicines that combines biological and chemical components into a modular nanometric drug delivery system. The core of the system is a filamentous phage particle that is produced in the bacterial host *Escherichia coli*. Target specificity is provided by a targeting moiety, usually an antibody that is displayed on the tip of the phage particle. A large drug payload is chemically conjugated to the protein coat of the phage via a chemically or genetically engineered linker that provides for controlled release of the drug after the particle homed to the target cell. Recently we have shown that targeted drug-carrying phage nanomedicines can be used to eradicate pathogenic bacteria and cultured tumor cells with great potentiation over the activity of the free untargeted drug. We have also shown that poorly water soluble drugs can be efficiently conjugated to the phage coat by applying hydrophilic aminoglycosides as branched solubility-enhancing linkers.

**Results:**

With an intention to move to animal experimentation of efficacy, we tested anti-bacterial drug-carrying phage nanomedicines for toxicity and immunogenicity and blood pharmacokinetics upon injection into mice. Here we show that anti-bacterial drug-carrying phage nanomedicines that carry the antibiotic chloramphenicol conjugated via an aminoglycoside linker are non-toxic to mice and are greatly reduced in immunogenicity in comparison to native phage particles or particles to which the drug is conjugated directly and are cleared from the blood more slowly in comparison to native phage particles.

**Conclusion:**

Our results suggest that aminoglycosides may serve as branched solubility enhancing linkers for drug conjugation that also provide for a better safety profile of the targeted nanomedicine.

## Background

The majority of known anti-bacterial approaches are based on the selectivity and potency of the antibiotic molecule itself, excluding highly toxic and non-specific therapeutics from the clinical application. The attaching of a non-selective drug to the suitable targeted carrier could provide specificity to the therapeutic complex and may improve its physical and biological characteristics such as solubility, cytotoxicity, circulation half-life and distribution to certain tissues and cells.

This study continues the evaluation of an anti-bacterial approach we have recently introduced that included the application of the bacteriophage (phage) nanoparticle as targeted, high-capacity anti-bacterial drug carriers [1,2]. In this approach, the phage particle served as a drug-carrying platform that was genetically and chemically modified to display a targeting moiety (mostly an antibody) on its surface and was used to deliver a large payload of a cytotoxic drug to the target bacteria. The platform was based on the f1 filamentous coliphage that was displaying anti-bacterial peptide or antibody on the minor pIII coat protein. The displayed protein provided the specific targeting to the pathogen while the natural host specificity of the phage was not relevant to its therapeutic potential. The cytotoxic drug (chloramphenicol) was chemically modified to contain an esterase cleavage-susceptible linker and was chemically conjugated to phage via a hydrophilic linker (the aminoglycoside neomycin). The controlled (or in fact, delayed) drug release was facilitated by serum esterases activity. The targeted drug-loaded phage particle demonstrated its ability to specifically recognize several model bacterial pathogens and to inhibit their growth *in vitro *by creating the high local drug concentration near the target bacteria. The carrying capacity of phage particle was established as 10,000 chloramphenicol molecules per phage and the improvement factor of 20,000 in drug potency in comparison to the free drug had been observed [2].

When particulate nanoparticles are considered for *in vivo *application, issues of pharmacokinetics, toxicity and immunogenicity become relevant [3-5]. The objectives of this study were to evaluate the *in vivo *characteristics of targeted drug-carrying phage nanomedicines such as toxicity, immunogenicity and pharmacokinetics and to estimate the potential of phage-based drug-carrying approach for *in vivo *application.

## Results and discussion

### Basic properties of Neo-CAM conjugated phages vs. native phages

**Figure 1:**
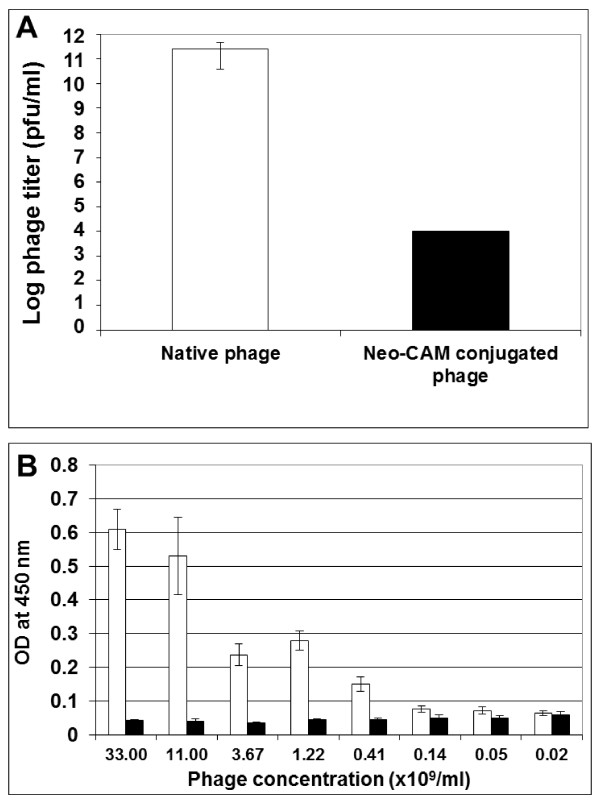
**The phage basic properties are influenced by drug conjugation process**. (A) The phage infectivity evaluation prior vs. post drug conjugation by live titration. *E. coli *bacteria (TG-1 strain) were used for infectivity determination. (B) The determination of the phage recognition by monoclonal anti-pVIII antibodies by ELISA. White bars represent native phages, black bars represent Neo-CAM conjugated phages.

Conjugation of the drug chloramphenicol to the phage coat proteins involves EDC (1-ethyl-3-(3-dimethylaminopropyl) carbodiimide) chemistry that is used to couple primary amines provided by the neomycin-chloramphenicol prodrug to surface-exposed carboxyl groups on the phage coat proteins. We found that the drug conjugation process that affected all surface exposed coat proteins radically influenced the basic phage characteristics - its ability to infect its natural bacterial host. As shown in Figure 1, following Neo-CAM conjugation, the phages almost totally lost their infectivity, with less than 0.00001% of drug carrying phages kept its ability to infect its host (*E. coli*). This can be regarded as an advantage, as one of the concerns in classical phage therapy is that once introduced into the body that (infective) phages may undergo replication cycles resulting in their number becoming uncontrollable. In addition, the antigenic profile of the phages was attenuated as well, as the drug conjugation presumably affected the epitopes on the phage surface that prevented the phage recognition by a monoclonal anti-PVIII antibody (Figure 1B). These were early indications that dramatic changes in basic phage properties occurred following drug conjugation to the surface of the phage particles, which may influence their *in vivo *characteristics upon injection into animals, such as toxicity and immunogenicity.

### Neo-CAM carrying phages have low toxicity in vivo

**Figure 2:**
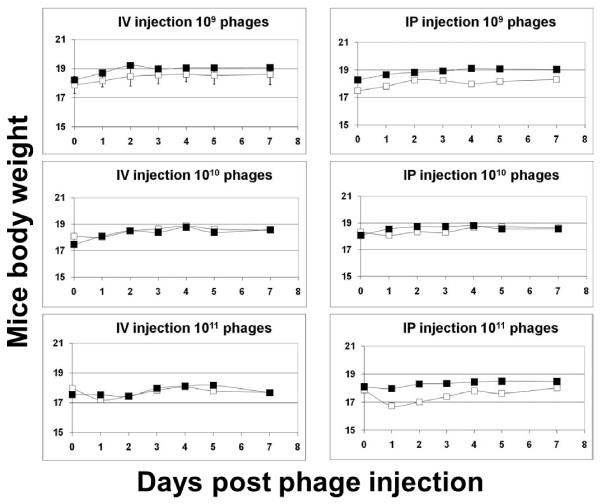
**Toxicity evaluation of Neo-CAM conjugated phages**. The open squares refer to native (unconjugated) phages, the filled squares refer to Neo-CAM carrying phages. The single dosage of examined phages were injected intravenously (IV) or intraperitoneally (IP) at 10^9 ^phage/dose (A, B), 10^10 ^phage/dose (C, D), 10^11 ^phage/dose (E, F). The average corresponds to 5 mice per group.

Previous studies on phage therapy demonstrated that when prepared properly, phage injection does not harm animals or humans upon oral administration [6] and minimal toxicity was detected in mice upon i.v. administration to mice [7]. However, drug-carrying phages were not evaluated in vivo before. We evaluated the toxicity of Neo-CAM carrying bacteriophages to evaluate the effect of conjugation of the drug with the serious toxic characteristics. The mice received a single injection of either native or of Neo-CAM conjugated phages at three concentrations (10^9 ^(low), 10^10 ^(medium) and 10^11 ^(high) phage per dose). The intravenous as well as intraperitoneal administration routes were examined. The mice weight and behavior were observed for 8 days followed the injection. During the observation period the mice showed no sign of distress or toxicity (no deaths were recorded), and no weight loss nor behavioral changes as a result of low and medium dose injections. The high dose (10^11 ^phages) caused up to 2% and 6% weight loss by Neo-CAM phage and by native phage, respectively. Considering that the conjugated drug - chloramphenicol has been reported as haemolytic upon systemic injection [8], it was reassuring to observe that the drug conjugated phages did not demonstrate drug-related toxicity and even showed loss toxicity at high dose in comparison to un-conjugated phages (Figure 2).

### Neo-CAM carrying phages are less immunogenic than unconjugated phages

**Figure 3:**
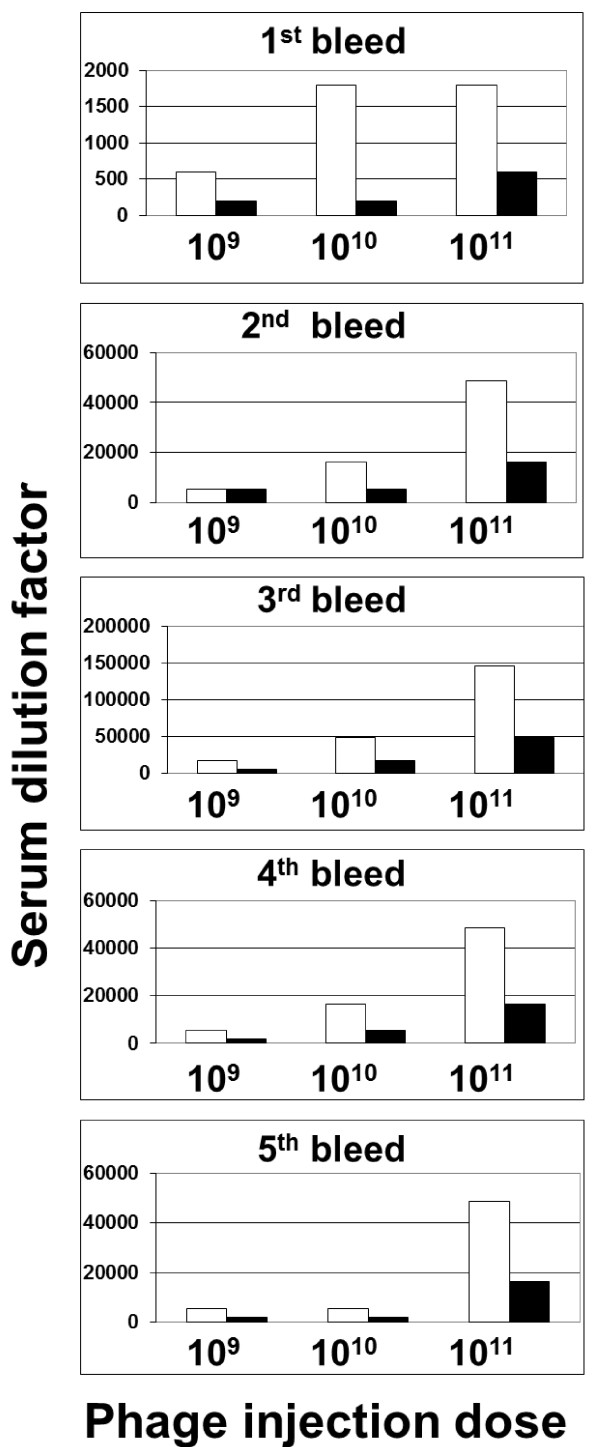
**ELISA analysis of anti-phage antibody titer in mice serum**. BALB/c mice were immunized with native or with Neo-CAM carrying phages by intravenous (IV) injection. Three doses were evaluated: low (10^9 ^phage/dose), medium (10^10 ^phage/dose) and high (10^11 ^phage/dose). The sera samples were collected a week following each injection (weeks 1, 3 and 5), and on the weeks 10 and 22 (n = 5 mice per group). Anti-phage serum titers were determined by ELISA where serial serum dilutions were applied onto phage-coated ELISA plate and detected using HRP-conjugated goat anti mouse antibodies.

**Figure 4:**
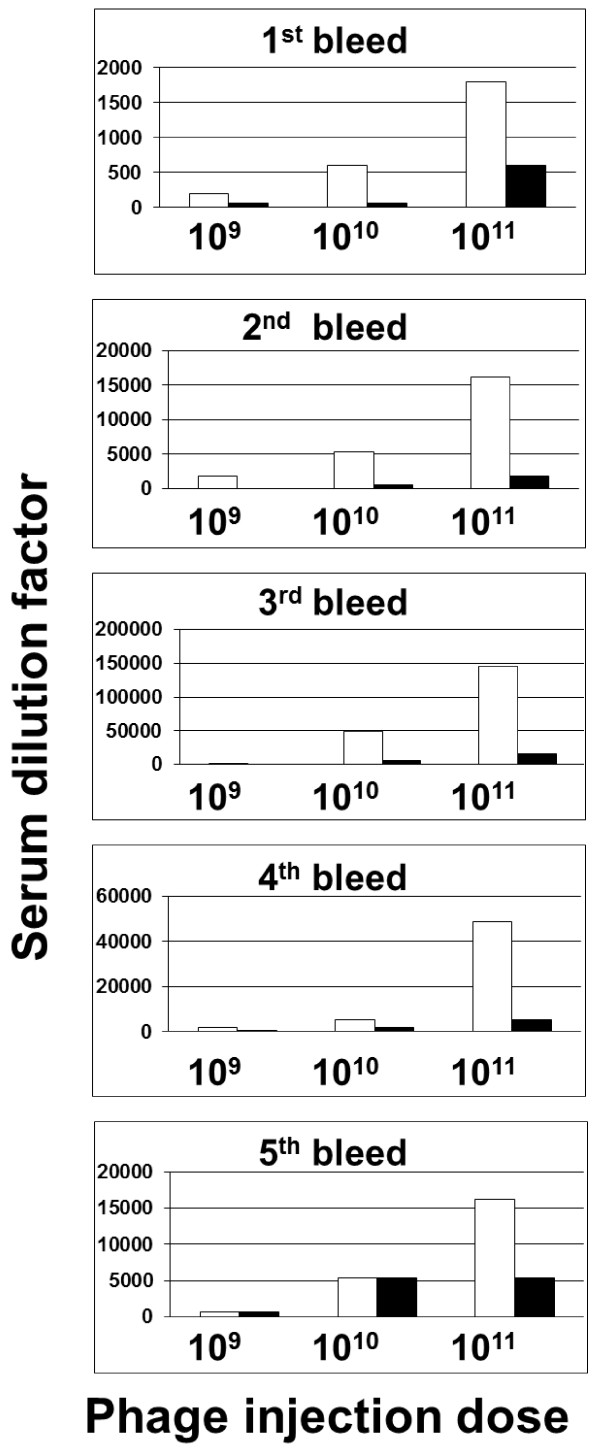
**ELISA analysis of anti-phage antibody titer in mice serum**. BALB/c mice were immunized with native/Neo-CAM carrying phages by intraperitoneal (IP) injection. Three doses were evaluated: low (10^9 ^phage/dose), medium (10^10 ^phage/dose) and high (10^11 ^phage/dose). The sera samples were collected a week following each injection (weeks 1, 3 and 5), and on the weeks 10 and 22 (n = 5 mice per group). Anti-phage serum titers were determined by ELISA where serial serum dilutions were applied onto phage-coated ELISA plate and detected using HRP-conjugated goat anti mouse antibodies.

The high immunogenicity of phages is a well-known feature that limits the rapid development of phage-based therapies. In particular, filamentous phage are highly immunogenic, a property that was used for their application as carriers and boosters for vaccination purposes [9-11]. High immunogenicity is counter-productive for a drug carrying nanomedicine destined to circulate in the body. In order to evaluate the immunogenic properties of drug-carrying phages, BALB/c mice were immunized intravenously or intraperitonealy with 10^9 ^(low), 10^10 ^(medium) or 10^11 ^(high) Neo-CAM carrying or native phages per dose. Three injections were performed at two weeks intervals. The mice sera were examined for the presence and titer of anti-phage antibodies by ELISA. As shown in Figures 3 and 4 mice that were injected with drug-carrying Neo-CAM phages showed much lower titers of anti-phage antibodies than the mice received native phages. As shown in Figure 3, when phages were administered IV, serum anti phage titer rose in a dose dependent manner and reached a maximum after the third injection (a titer of 150000 for unconjugated and 40000 for Neo-CAM carrying phages, a four-fold difference at the maximal injected dose). Serum anti phage titer declined after phage injection was completed, but remained significant up to six months after the first injection (a titer of 50000 for unconjugated and 16000 for Neo-CAM carrying phages at the maximal injected dose). For the groups that were injected with fewer phages, the fold differences in serum titer were similar while the titers themselves were lower than the ones reached at the maximal dose.

As shown in Figure 4, when phages were administered IP, the trend was similar but the titers were lower than those received after injecting similar phage doses to the mice IV. Serum anti phage titer rose in a dose dependent manner and reached a maximum after the third injection (a titer of 150000 for unconjugated and 15000 for Neo-CAM carrying phages, a ten-fold difference at the maximal injected dose). Serum anti phage titer declined after phage injection was completed, but remained significant up to six months after the first injection (a titer of 16000 for unconjugated and 5000 for Neo-CAM carrying phages injected at the maximal dose). For the groups that were injected with fewer phages, the fold differences in serum titer were similar, with an exception that sera obtained during the two last bleeds at low and medium injection doses showed low titers with negligible differences between sera from mice that were injected with unconjugated phages in comparison to mice that were injected with Neo-CAM carrying phages.

**Figure 5:**
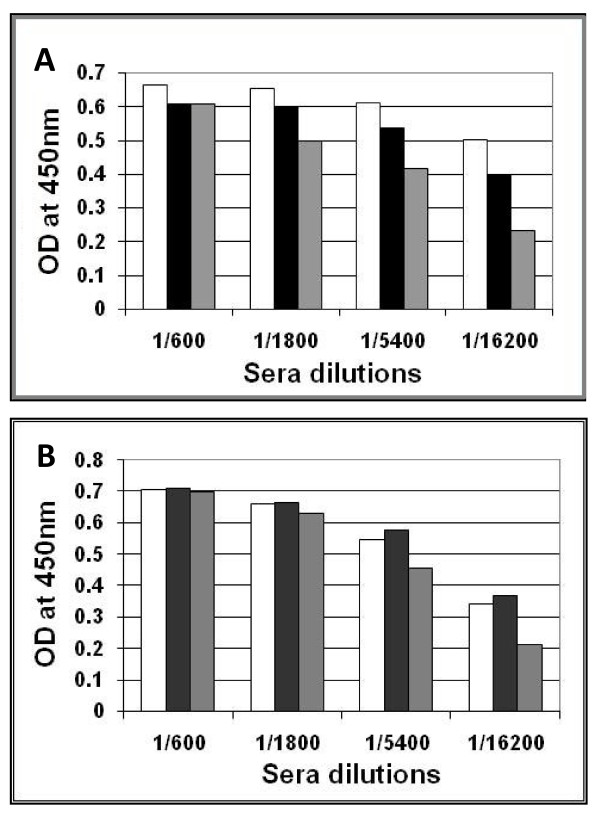
**ELISA analysis of anti-drug immune response in BALB/c mice immunized with native (A) or Neo-CAM carrying (B) phages**. Sera samples were obtained from mice immunized with the highest phage concentration (10^11 ^phage/dose) following the two boost injections and examined on their ability to bind native (white), Neo-CAM conjugated (black) or Neo conjugated (grey). Neither anti-chloramphenicol nor anti-neomycin antibodies were detected in sera of immunized mice. The average corresponds to 5 mice per group.

To evaluate the presence of anti-drug antibodies the sera samples that were collected from mice immunized with Neo-CAM carrying phages, a different ELISA was carried out. Sera samples were examined for their ability to bind native, neomycin-chloramphenicol conjugated and neomycin only conjugated phages. We found that sera obtained from mice that were immunized with native phages did not show preference in binding native or drug-conjugated phages (Figure 5A), thus suggesting there was no difference in phage adsorption to the ELISA plate. The next step was to examine sera of mice that were immunized with Neo-CAM carrying phages. The assay demonstrated that antibodies presented in mice sera samples were able to recognize conjugated as well as un-conjugated phages to the same extent (Figure 5B) suggesting that no specific anti-drug antibodies were raised in the mice.

An additional concern could be the immunogenicity of the targeting antibody. While not evaluated experimentally in this study, there a well-established solution for that, of using humanized or human antibodies for targeting.

The targeted drug-carrying phages, first introduced in 2006 [1] are a powerful tool for selective eradication of pathogenic bacteria. Our group has demonstrated the targeting ability of phage nanoparticle that was provided by display of bacteria-specific peptide or antibody on the phage surface. The conjugation of non-selective drug to the targeted phage carrier via a labile linker enabled the drug accumulation and action near the target bacteria that resulted in severe bacteria growth inhibition *in vitro *[2]. Published in vivo phage therapy studies may provide us with preliminary data concerning the behavior of phages following *in vivo *administration. However, while conventional research of phage therapy could help us to elucidate the main principles of phage in vivo behavior, there are many differences between "classic" phage therapy and drug carrying approach that may influence the phage behavior in animals and humans. Earlier studies in classical phage therapy and also in filamentous phage-based immunization protocols established phages as a safe and non-toxic [9,12-14]. However, the drug our phage carry (chloramphenicol) is not allowed in systemic administration because of its side effects [8], raising concern that drug-mediated toxicity may be a limitation of our system. The toxicity evaluation of Neo/CAM carrying phage we carried out showed that even the highest concentration of 10^11 ^phage/dose did not cause a significant toxic effect. We assume that chloramphenicol lost its toxic properties as a result of the conjugation, converting it into a "prodrug" state as long as it is conjugated to the phage carrier. In addition, we calculated that even complete release of the conjugated drug yields, even with clearance rate neglected, a systemic concentration that is too low to produce a harmful effect. This in general is an advantage of a targeted drug-delivery system over non-targeted ones.

The next subject of concern was the phage immunogenicity. Filamentous phages were known as highly immunogenic and had been used as vaccine carries while the vaccination with peptide-displaying phage raised a high antibody titer against the displayed molecule and more so, the phage itself [15]. The immunogenic properties are probably caused by the multiple repeats of pVIII protein on the phage surface and a large size of a phage particle. Drug conjugation by EDC chemistry targets the free carboxyl groups on phage proteins that extremely influence the phage physical and biological features. For example, EDC treated phages cannot be recognized by commercial monoclonal anti-phage antibodies, cannot be precipitated by PEG/NaCl, and mostly lose their infectivity as demonstrated above. Our results suggest that Neo-CAM carrying phages were far less immunogenic in comparison to native phages. The repeated injections of drug-conjugated phages resulted in reduced anti-phage antibody titer (50,000 for intravenous and 18,000 for intraperitoneal injection), while the antibody titer for un-conjugated phage injections reached 150,000. The sera samples that were collected 1.5 and 5 months after the last injection reported the extinguishing of anti-phage titer for both conjugated and un-conjugated treatments. The intraperitoneal administration demonstrated lower absolute titers for drug-carrying drug in comparison to native phage, suggesting this administration route may be more appropriate for further study. Based on the described results, we propose that the reduced immunogenicity of drug-carrying phage treatment was achieved due to neomycin, used for chloramphenicol conjugation. Neomycin was chosen as a hydrophilic agent that can mediate between the phage platform and hydrophobic drug (chloramphenicol). The conjugation of this aminoglycoside antibiotic probably provides the "sugar envelope" for a phage particle turning it into much less immunogenic. In addition, the immunization of BALB/c mice with directly conjugated drug (without aminoglycoside linker) resulted in induced anti-phage antibody titer similar to un-conjugated phage treatment (our unpublished results). It is widely known that sugars and amino-related coating (such as PEGylation and poly(amino acid)s conjugation) of immunogenic protein reduce the immune response against it and improve its pharmacokinetic features [16-19]. We suggest that aminoglycoside conjugation to proteins provides its "shielding" from the immune system similar to these reported effects. In addition, we demonstrated that the drug presentation on the phage carrier did not lead to anti-drug immune response against neomycin or chloramphenicol. We propose that one or both of the following models may account for the reduced immunogenicity of targeted Neo-Cam carrying phages:

Chloramphenicol molecules are slowly released from the phage surface by serum esterases. As a result, the phage is constantly changing and does not present a continuing stimulus to the immune system that as a result gets "confused and unfocused".

Similarly to reduction of immunogenicity of biopharmaceuticals by PEG [20], neomycin remains on the phage surface and as it was implied above, it might shield the phage particle from the immune system.

The reduced immunogenicity of drug-carrying phages should allow a number of sequential treatments (repeat therapy) without severe side effects that is highly significant for anti-bacterial treatments.

### Comparative pharmacokinetics of Neo-CAM carrying phage and native phage in mice serum

Previous studies on native filamentous phage pharmacokinetic properties in mice demonstrated rapid removal of phage particles from the circulation 5 to 15 min following intravenous injection [21]. Another study suggested that bacteriophages' half-life in mouse serum is about 4 hours [22]. To determine the half-life of drug-carrying phage the BALB/c mice were injected with either native or Neo-CAM carrying phages and blood samples were taken at varying time points following injection. In order to evaluate the phage particles concentration in the samples, two different approaches were chosen: CFU recovery and Real-Time PCR.

**Figure 6:**
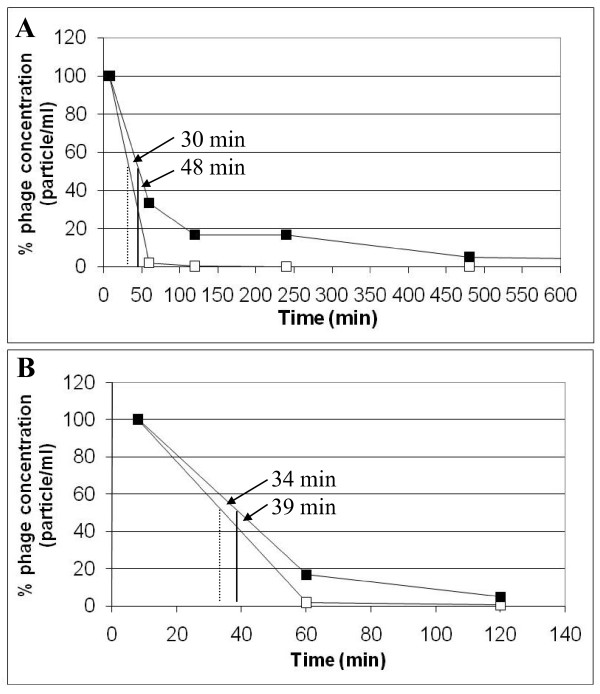
**Analysis of Neo-CAM carrying vs. native phages blood pharmacokinetics in mice: recovery of colony forming units (CFU) (A) or phage DNA quantification by Real-Time PCR (B)**. BALB/c mice were injected with 10^11 ^native/Neo-CAM carrying phages in 200 μL sterile PBS and blood samples were collected at the indicated time points. (A) For CFU determination the serum samples were incubated with *E. coli *bacteria, and then plated on agar supplemented with appropriate antibiotics and colony forming units counted the next day. (B) For phage DNA quantification the serum samples were amplified by Real-Time PCR and phage DNA amount was calculated using the calibration curve. The % phage concentrations were calculated relatively to the phages found at the 8 min time point.

CFU recovery is widely used for phage quantification while the phage amount is calculated corresponding to infective phage particles. Bacteriophages following the drug conjugation by EDC chemistry partially lose their infectivity, thus complicating interpretation of the results. According to the phage quantification based on colony forming units (CFU) recovery, Neo-CAM carrying phages remain in the bloodstream with a half life of 48 min (almost 60% more the time of native phages) (Figure 6A). The total clearance of phage particles from blood circulation required up to 24 hours. This result further suggests that Neomycin "sugar coating" of the phage nanoparticles may serve as an efficient anti-immunogenic agent, which can reduce immune response, by shielding a nanoparticle (liposomes or phage) from both the immune system and the RES system which is primarily responsible for removal of phages from the circulation [22].

To confirm the previous result, Real-Time PCR was conducted to quantify phage DNA in serum samples. While the phage infectivity was damaged during the drug conjugation process, the phage DNA remained unaffected. We developed a Real-Time PCR assay for phage DNA quantification in serum samples as an accurate method for determination of phage DNA concentration. Our results suggest that Neo-CAM phages remain longer in the circulation than the unconjugated phages. The differences (39 min for Neo-CAM carrying phage in comparison to 34 min for native phage particles (Figure 6B)) were similar but not identical to those found by CFU determination. These results further demonstrated that Neo-CAM conjugation to phage coat proteins influenced the phages' pharmacokinetic properties and increased its serum half-life in mice.

It is intriguing that the immunogenicity of Neo-CAM-conjugated phages is reduced compared to native phages while their serum half-life in increased. Presently it is not to what extent anti-phage antibodies rise as a result of direct stimulation of phage-specific B-cells, or processing and presentation of phage epitopes on professional antigen-presenting cells (APCs) (such as the macrophages and dendritic cells that are part of the RES system that removed phages from the body) plays a dominant role. Our results may suggest that APCs play a key role in the development of anti-phage antibodies, and the "sugar coating" that causes the Neo-Cam-carrying phages to be removed with different kinetics also attenuate antigen presentation and antibody production. At this time, such an assumption is highly speculative.

The evaluation of *in vivo *activity of targeted drug-carrying phages would be the next determining step in technique development. Our preliminary results on *Staphylococcus aureus *bacteria systemic infection of BALB/c mice demonstrated the partial recovery followed by single injection of anti-*S. aureus *Neo-CAM carrying phages in comparison to 100% lethality in control group (unpublished data).

## Conclusion

There is a renaissance of interest in the antimicrobial potential of phages as more pathogens become multiply antibiotic resistant [23]. While most efforts in the field of phage therapy use "classical" approached where the target bacteria are killed by virtue of the phage acting as a natural killer of the bacterial host, the approach of targeted drug-carrying phage nanomedicines we pioneered is different. Our phages are rendered non-infective by the drug conjugation chemistry, and the natural phage-host specificity is replaced by the target specificity conferred by the targeting moiety that is displayed on the phage. Still, any therapeutic application of phages will have to involve mitigation of harmful properties such as toxicity and immunogenicity. Our study demonstrated that drug-carrying phages, and in particular phages that carry chloramphenicol via an aminoglycoside linker were safe and feebly immunogenic in a small animal model and would be able to continue its way to therapy. Future work will focus on the targeting improvement which is crucial for the potency and selectivity of the conjugated drug. The other research direction can be the finding an alternative drug release method based on contact with treated pathogen that can greatly improve the therapeutic activity of the approach *in vitro *as well as *in vivo*.

## Methods

### Phage preparation

For propagation of fUSE5 filamentous phage, a phage infected *E. coli *(DH5α strain) colony was inoculated into 0.5 liter of 2 × YT medium supplemented with 12.5 μl/ml tetracycline and grown overnight with shaking (250 rpm) at 30°C. The culture was centrifuged and filtered (to eliminate remaining bacteria) through a 0.45 μm vacuum filter device (Amicon, USA). 1/5 volume of PEG/NaCl (20% polyethylene 6000, 2.5 M NaCl) was added to the supernatant, mixed well and incubated overnight at 4°C for phage precipitation. The phage-precipitates were collected by centrifugation at 6000 rpm for 30 min at 4°C, resuspended in sterile water at a final concentration of 5 × 10^13 ^PFU/ml, filtered and stored at 4°C.

### Drug conjugation

The chloramphenicol prodrug was synthesized and conjugated to the phage particles via a neomycin linker as described [2]. The procedure included drug conjugation using EDC chemistry that provided the coupling of the exposed carboxyl side chains on the phage coat and neomycin primary amine. For removal of free drug excess and reduction of endotoxin content, the un-conjugated as well as the drug conjugated phages were dialyzed by two sequential steps against 1000 volumes of 0.3 M NaCl at 4°C for 16 hours each. Phages that carry chloramphenicol via a neomycin linker are referred to as "Neo-CAM phages".

### Phage quantification by live titration

DH5αF' strain of *E. coli *bacteria were grown in 2 × YT media till A_600 nm _reached 0.8. Then 90 μl of bacteria were infected with 10 μl phage dilutions and incubated 1 hour at 37°C. Drops of 10 μl of incubated bacteria were plated onto agar plates supplemented with 12.5 μl/ml tetracycline and grown at 37°C overnight to develop colonies of phage infected cells. Phage quantity was calculated according to the resistant bacteria colonies number multiplied by phage dilution in the drop.

### Mouse toxicity evaluation

Animal experimentation was carried out with approval of the Tel-Aviv University IRB. Female BALB/c mice (8-10 weeks old, ~20 gr, 5 mice in each group) were given a single injection intravenously (tail vein) or intraperitonealy of Neo-CAM phage or native (naked) phage. The phages were diluted in phosphate buffer saline (PBS) to 10^9^, 10^10 ^or 10^11 ^phages/dose and injected in total volume of 200 μl. Mice were monitored for weight loss or death for 8 days after infection.

### Immunogenicity assay

Female BALB/c mice (8-10 weeks old, ~20 gr, 5 mice per group) were immunized with 10^9^, 10^10 ^or 10^11 ^Neo-CAM conjugated or native phages per dose. Phages were diluted to the desired concentration in phosphate buffered saline (PBS) in a final volume of 200 μl. Two routes of administration were evaluated: intravenously (tail vein) and intraperitonealy. Mice received the injections 3 times on weeks: 0, 2, 4 and were bleed from the orbital vein on the weeks: 1, 3, 5, 10 and 22. The blood samples were clotted on ice for 1 hour, than centrifuged and sera were collected and stored at -20°C.

### ELISA

Drug-conjugated (Neo-CAM) and unconjugated phages were evaluated for their recognition by monoclonal anti-fd antibodies. For that purpose, 96-well ELISA plate was coated with polyclonal mouse anti-phage serum (diluted 1:1500 in PBS) for 1 hour at 37°C and blocked with 3% skim milk in PBS for 1 hour at 37°C. The plate was washed with PBS containing 0.05% (v/v) tween 20 (PBST) and incubated with Neo-CAM conjugated or native phage diluted in PBST. Following 1 hour incubation at room temperature, the plates were washed three times with PBST and incubated with an HRP-conjugated monoclonal mouse-anti-PVIII antibody (diluted 1:5000 in PBST) for 1 hour at room temperature. Followed by 3 times wash in PBST, the plates were developed with the chromogenic substrate TMB and color development was terminated with 1 M H_2_SO_4_. The plates were read at 450 nm.

For determination of anti-phage antibody titer in sera of immunized mice, the 96-well ELISA plate was coated with 10^10 ^native phage suspension in PBS/well for 2 hours 37°C followed by blocking with 3% skim milk in PBS overnight at 4°C. All subsequent steps were performed at room temperature. Dilutions (from 1:200) of sera samples in PBS were applied onto plates and incubated for 1 hour. The plates were washed three times with PBST, followed by incubation with an HRP-conjugated goat-anti-mouse antibody (diluted 1:5000 in PBST) for 1 hour, than washed 3 times in PBST. The plates were developed as described above.

In order to estimate the presence of anti-drug antibodies that may have been raised during the mice immunization, 96-well ELISA plates were coated with 10^10 ^phage suspension in PBS/well of the following phages: native (un-conjugated), Neo-CAM carrying, or Neo only carrying for 2 hours at 37°C. Plates were blocked with 3% skim milk in PBS at 4°C overnight followed by three washes in PBST. 3-fold dilutions (from 1:600) of sera samples (obtained from mice immunized with the highest (10^11 ^phages/dose) intravenous injections of Neo-CAM carrying and native phages) in PBS were applied onto plate and were incubated for 1 hour. The following steps were executed exactly as the previously described assay.

All the ELISA experiments were carried out in duplicates or triplicates at least 3 independent times during the study.

### Determination of Neo-CAM phage vs. native phage circulation half life time in mice

To evaluate and compare the blood pharmacokinetic properties of Neo-CAM phage and native phage (that do not carry drug), female BALB/c mice (8-10 weeks old, ~20 g, 5 mice in group) were given a single intravenous dose of 10^11 ^Neo-CAM/native phage by injection into the tail vein. Blood samples were collected from the orbital vein at 8, 60, 120, 240 and 480 min and 24 hours after injection. Each mouse was bled two or three times, so different mice were used to collect data for the various time points. Subsequent to clotting the blood samples on ice the serum concentrations of phages were determined by CFU recovery (live titration) or Real-Time PCR.

### Colony forming units (CFU) quantification in mouse serum

For CFU determination, 90 μL of F' *E. coli *TG-1 cells were infected with 10 μL of mouse serum dilutions. The infected bacteria were incubated 1 hour at 37°C and then plated on agar plate supplemented by 20 μg/ml tetracycline. The number of tetracycline-resistant colonies or colony forming units (CFU) was counted the next day. Phage concentration was calculated according to CFU number in the serum dilution.

### Phage quantification by Real-Time PCR

Real-Time PCR was used to quantify the phage DNA particles in serum. Real-Time PCR was performed as follows, 1 μL of serum diluted 1:100 in PBS, 500 nM fUSE5-RT (5'- CTTTGAACGAGGACAGATGC -3') and P5-*BsrGI*-For (5'- TCGTCAGGGCAAGCCTTATTC -3') primers, qPCR Supermix-UDG (containing: SYBR Green I fluorescent dye, platinum TaqDNA polymerase, MgCl_2_, dNTPs, uracil-DNA glycosylase (UDG), reaction buffer) (Invitrogen). The reaction conditions were: 95°C for 2 min, 45 cycles of 95°C for 15 sec and 60°C for 30 sec, final extension 60°C for 5 min. The reaction was carried out and analyzed as recommended by the supplier (Applied Biosystems). Fluorescence data were collected at the end of each 60°C annealing step. The sample was designated positive when there was an exponential increase in fluorescence during the first 30 cycles of PCR amplification (crossing point, ≤ 30). Samples with crossing points of > 30 cycles were determined as < 10^6 ^phage/ml. Phage concentration in a sample was determined according to a calibration curve of Neo-CAM/native phage at increasing concentrations (10^6^-10^11 ^phage/ml).

## Competing interests

The authors declare that they have no competing interests.

## Authors' contributions

LV and IB designed the experiments. LV performed the experiments. Both authors wrote and approved the manuscript.
